# Population-Based Active Surveillance for Culture-Confirmed Candidemia — Four Sites, United States, 2012–2016

**DOI:** 10.15585/mmwr.ss6808a1

**Published:** 2019-09-27

**Authors:** Mitsuru Toda, Sabrina R. Williams, Elizabeth L. Berkow, Monica M. Farley, Lee H. Harrison, Lindsay Bonner, Kaytlynn M. Marceaux, Rosemary Hollick, Alexia Y. Zhang, William Schaffner, Shawn R. Lockhart, Brendan R. Jackson, Snigdha Vallabhaneni

**Affiliations:** ^1^Epidemic Intelligence Service, Division of Scientific Education and Professional Development, Center for Surveillance, Epidemiology, and Laboratory Services, CDC; ^2^Division of Foodborne, Waterborne, and Environmental Diseases, National Center for Emerging and Zoonotic Infectious Diseases, CDC; ^3^Department of Medicine, Emory University School of Medicine, Atlanta, Georgia; Atlanta Veterans Affairs Medical Center, Atlanta, Georgia; ^4^Maryland Emerging Infections Program, Johns Hopkins Bloomberg School of Public Health, Baltimore, Maryland; ^5^Acute and Communicable Disease Prevention, Oregon Health Authority, Portland, Oregon; ^6^Vanderbilt University School of Medicine, Nashville, Tennessee

## Abstract

**Problem/Condition:**

Candidemia is a bloodstream infection (BSI) caused by yeasts in the genus *Candida*. Candidemia is one of the most common health care–associated BSIs in the United States, with all-cause in-hospital mortality of up to 30%.

**Period Covered:**

2012–2016.

**Description of System:**

CDC’s Emerging Infections Program (EIP), a collaboration among CDC, state health departments, and academic partners that was established in 1995, was used to conduct active, population-based laboratory surveillance for candidemia in 22 counties in four states (Georgia, Maryland, Oregon, and Tennessee) with a combined population of approximately 8 million persons. Laboratories serving the catchment areas were recruited to report candidemia cases to the local EIP program staff. A case was defined as a blood culture that was positive for a *Candida* species collected from a surveillance area resident during 2012–2016. Isolates were sent to CDC for species confirmation and antifungal susceptibility testing. Any subsequent blood cultures with *Candida* within 30 days of the initial positive culture in the same patient were considered part of the same case. Trained surveillance officers collected clinical information from the medical chart for all cases, and isolates were sent to CDC for species confirmation and antifungal susceptibility testing.

**Results:**

Across all sites and surveillance years (2012–2016), 3,492 cases of candidemia were identified. The crude candidemia incidence averaged across sites and years during 2012–2016 was 8.7 per 100,000 population; important differences in incidence were found by site, age group, sex, and race. The crude annual incidence was the highest in Maryland (14.1 per 100,000 population) and lowest in Oregon (4.0 per 100,000 population). The crude annual incidence of candidemia was highest among adults aged ≥65 years (25.5 per 100,000 population) followed by infants aged <1 year (15.8). The crude annual incidence was higher among males (9.4) than among females (8.0) and was approximately 2 times greater among blacks than among nonblacks (13.7 versus 5.8). Ninety-six percent of cases occurred in patients who were hospitalized at the time of or during the week after having a positive culture. One third of cases occurred in patients who had undergone a surgical procedure in the 90 days before the candidemia diagnosis, 77% occurred in patients who had received systemic antibiotics in the 14 days before the diagnosis, and 73% occurred in patients who had had a central venous catheter (CVC) in place within 2 days before the diagnosis. Ten percent were in patients who had used injection drugs in the past 12 months. The median time from admission to candidemia diagnosis was 5 days (interquartile range [IQR]: 0–16 days). Among 2,662 cases that were treated in adults aged >18 years, 34% were treated with fluconazole alone, 30% with echinocandins alone, and 34% with both. The all-cause, in-hospital case-fatality ratio was 25% for any time after admission; the all-cause in-hospital case-fatality ratio was 8% for <48 hours after a positive culture for *Candida* species*.* *Candida albicans* accounted for 39% of cases, followed by *Candida glabrata* (28%) and *Candida parapsilosis* (15%). Overall, 7% of isolates were resistant to fluconazole and 1.6% were resistant to echinocandins, with no clear trends in resistance over the 5-year surveillance period.

**Interpretation:**

Approximately nine out of 100,000 persons developed culture-positive candidemia annually in four U.S. sites. The youngest and oldest persons, men, and blacks had the highest incidences of candidemia. Patients with candidemia identified in the surveillance program had many of the typical risk factors for candidemia, including recent surgery, exposure to broad-spectrum antibiotics, and presence of a CVC. However, an unexpectedly high proportion of candidemia cases (10%) occurred in patients with a history of injection drug use (IDU), suggesting that IDU has become a common risk factor for candidemia. Deaths associated with candidemia remain high, with one in four cases resulting in death during hospitalization.

**Public Health Action:**

Active surveillance for candidemia yielded important information about the disease incidence and death rate and persons at greatest risk. The surveillance was expanded to nine sites in 2017, which will improve understanding of the geographic variability in candidemia incidence and associated clinical and demographic features. This surveillance will help monitor incidence trends, track emergence of resistance and species distribution, monitor changes in underlying conditions and predisposing factors, assess trends in antifungal treatment and outcomes, and be helpful for those developing prevention efforts. IDU has emerged as an important risk factor for candidemia, and interventions to prevent invasive fungal infections in this population are needed. Surveillance data documenting that approximately two thirds of candidemia cases were caused by species other than *C. albicans*, which are generally associated with greater antifungal resistance than *C. albicans*, and the presence of substantial fluconazole resistance supports 2016 clinical guidelines recommending a switch from fluconazole to echinocandins as the initial treatment for candidemia in most patients.

## Introduction

Invasive candidiasis, caused by the yeast *Candida,* is one of the most common opportunistic fungal infections worldwide (*1*,*2*). Invasive candidiasis includes, among other manifestations, intra-abdominal infections, osteomyelitis, and bloodstream infections (candidemia), with candidemia being the most common type of invasive candidiasis. In the United States and elsewhere, *Candida* species are a leading cause of health care–associated bloodstream infections (*3*–*5*). Candidemia is associated with prolonged hospitalizations, high health care costs, substantial morbidity, and all-cause in-hospital mortality of up to 30% (*6*).

*Candida* is a common commensal organism of the gastrointestinal tract and can live on skin (*7*). Disruption of the normal barriers provided by the gastrointestinal tract or skin can lead to invasive infections (i.e., autoinfection). Overgrowth and translocation into the bloodstream can occur under the stressful physiologic conditions that generally occur during long-term hospitalizations and intensive care unit (ICU) stays. Recent abdominal surgery and other medical interventions, disruption of the microbiome from antibiotics, receipt of total parental nutrition (TPN), diabetes, malignancies, neutropenia, use of immunosuppressive therapies, and presence of indwelling catheters such as central venous catheters (CVCs) and other devices (*8*–*10*) are all risk factors for candidemia. Premature newborns with indwelling catheters also are at increased risk for candidemia (*11*–*13*). In addition to autoinfection, infections with certain species of *Candida*, particularly *Candida auris* and *Candida parapsilosis*, can result from transmission between patients in health care settings (*14*).

Underlying conditions that contribute to candidemia have changed over time as guidelines and practices for prophylactic antifungal therapy and CVC care have changed. For example, antifungal prophylaxis is now routinely used for extremely premature newborns in some neonatal units and for patients with certain types of hematologic malignancies, dramatically reducing rates of candidemia in these populations (*15*,*16*).

A few hundred species of *Candida* exist, a small proportion of which causes nearly all invasive infections in humans. *Candida albicans* is the most common species that causes candidiasis in the United States (*1*); however, the proportion of infections caused by species other than *C. albicans*, such as *Candida glabrata* and *C. parapsilosis*, has grown in the last few decades (*17*). These species exhibit higher levels of resistance to antifungal medications and might be associated with higher mortality than *C. albicans* (*18*). Recent reports indicate an increase in multidrug-resistant *C. glabrata* isolates in the United States (*19*,*20*). Equally concerning are newly emerging species of *Candida*, such as *C. auris*, which was first described in 2009 (*21*) and has since been reported in approximately 30 countries, including the United States (*22*). *C. auris* is resistant to multiple drugs and has caused large health care–associated outbreaks, spreading readily within certain health care facilities and creating a worldwide public health threat (*14*).

The incidence of candidemia in the United States has been measured periodically in different regions and populations. Incidence increased fivefold during 1980–1990, according to surveillance conducted as part of the National Nosocomial Infections Surveillance (NNIS) system (*23*,*24*). The incidence of candidemia started to decrease in the mid-1990s through the mid-2000s among low birthweight newborns, in part because of recommendations for fluconazole prophylaxis in certain settings (*25*–*27*). In population-based surveillance performed in the metropolitan areas of Atlanta, Georgia, and Baltimore, Maryland, candidemia incidence (primarily among adults) increased 10%–40% from the early 1990s and the late 2000s, which was followed by more recent reports of decreases in these areas (*6*,*28*–*30*).

Because of these changes, monitoring candidemia incidence in various populations, characterizing the distribution of species causing candidemia, estimating the prevalence of antifungal drug resistance, and determining whether risk factors, treatment, and outcomes for candidemia have changed over time are important. However, candidemia is not required to be reported in most states and is not a nationally notifiable disease, with the exception of *C. auris* infections (*31*), which are a small percentage of candidemia cases in the United States. Candidemia surveillance conducted through CDC’s Emerging Infections Program (EIP), a collaboration among CDC, state health departments, and academic partners that was initiated in 1995, is the only source of population-based information on candidemia in the United States (*32*). EIP surveillance for candidemia started in two sites (in Georgia and Maryland) in 2008 and expanded to two more sites (in Oregon and Tennessee) in 2011. This report includes 2012–2016 data from all four sites. The findings can be used by health care providers, infection control practitioners, stakeholders in the health care industry, and public health officials at federal, state, and local levels to promote awareness of candidemia incidence, risk factors, and outcome and to inform prevention measures.

## Methods

### Data Source 

During 2012–2016, CDC’s EIP (*32*) conducted active population-based surveillance for culture-confirmed candidemia in four sites: Georgia (eight counties in the metropolitan Atlanta area, with a 2014 population of 3.93 million), Maryland (city of Baltimore and Baltimore County, with a 2014 population of 1.45 million), Oregon (Portland tricounty area, with a 2014 population of 1.73 million), and Tennessee (nine counties surrounding Knoxville in East Tennessee, with a 2014 population of 943,000). The combined population under surveillance was approximately 8.06 million persons, representing approximately 2.5% of the U.S. population in 2014.

### Surveillance Case Definition

A case of candidemia was defined as a blood culture positive for a *Candida* species collected from a resident of the surveillance area during 2012–2016. An episode was defined as the 30-day period after the initial culture was positive. A new culture that was positive after the 30-day period was counted as a different case in the same patient. Any blood cultures positive for a *Candida* species within 30 days of the initial positive culture from the same patient were considered part of the same case, or episode, including different *Candida* species identified within the 30-day period or multiple *Candida* species found on the date of initial positive culture. The date of candidemia refers to the date the initial blood culture that yielded *Candida* was collected. Unless specified, data are presented at the case level because each of the measured exposure variables (e.g., time from hospital admission to culture) can change from case to case in the same person. However, demographic data are at the patient level because characteristics such as sex and race do not change from case to case in the same person. 

### Data Collection

Clinical, reference, and commercial laboratories that serve the population in the surveillance catchment areas were recruited to participate in the surveillance program and report cases of candidemia to the local surveillance officer. Once notified of a positive *Candida* blood culture, surveillance officers from each site used the surveillance case definition to determine case status and completed a standardized case report form to gather demographic and clinical data from the medical record. Surveillance officers received detailed instructions on completing the abstraction form and training in chart abstraction. In addition, surveillance officers performed periodic audits of laboratory microbiology records to ensure completeness of reporting. The corresponding *Candida* species isolates were sent to CDC for species confirmation and antifungal susceptibility testing. Deidentified data were sent to CDC.

### Variables Assessed

The chart review and case report forms used to collect data are available (2010–2013 long chart review form, https://stacks.cdc.gov/view/cdc/80195; 2010–2013 short chart review form, https://stacks.cdc.gov/view/cdc/80196; 2014 case report form, https://stacks.cdc.gov/view/cdc/80193; and 2016 case report form, https://stacks.cdc.gov/view/cdc/80194). The forms include information on demographic data, including age at time of positive culture, sex, and race. Adults were defined as patients aged >18 years. Other variables collected from medical chart review included underlying medical conditions and medical comorbidities; dates of hospital admission and discharge; receipt of antibiotics and antifungal medications; TPN in the 14 days before candidemia diagnosis; presence of a CVC within 2 days before diagnosis; treatment received for candidemia; and patient outcome (i.e., hospital discharge or death).

A candidemia case was defined as a health care–onset case when the initial positive *Candida* culture was obtained ≥3 days after admission; as a health care–associated community-onset case when the culture was obtained <3 days after admission for a patient with a recent health care exposure; or as a community-onset case when the culture was obtained <3 days after admission for a patient without a recent health care exposure. Recent health care exposure was defined as one or more of the following: residence in a nursing home, hospitalization in the 90 days before date of candidemia, or receipt of hemodialysis.

### Laboratory Methods

At CDC’s fungal reference laboratory, *Candida* species identification from isolates obtained from blood during 2012–2014 was performed using a Luminex assay or DNA sequencing of the D1/D2 subunit of the 28S ribosomal DNA (rDNA) (*33*). During 2015–2016, matrix-assisted laser desorption/ionization time of flight (MALDI-TOF) (*34*) was used for species identification. Antifungal susceptibility testing was performed at CDC with custom prepared microdilution plates (Trek Diagnostics) for fluconazole, voriconazole, anidulafungin, caspofungin, and micafungin according to the Clinical and Laboratory Standards Institute (CLSI) M27-A3 document guidelines (*35*). Growth was observed after 24 hours, and the minimum inhibitory concentration was determined by the lowest concentration of drug in which growth was decreased by approximately 50% compared with the control well. Isolates were categorized as resistant to each drug using the 2012 CLSI M27-S4 species-specific breakpoints (*36*). Amphotericin B susceptibility was tested using Etest strips (bioMérieux) according to the manufacturer’s instructions.

### Analysis

Crude candidemia incidence rates per 100,000 population are presented for each site by year. Percentages and age-, sex-, and race-specific incidence rates are presented for demographic characteristics of patients with candidemia during 2012–2016. Denominators used to calculate incidence rates for each surveillance site or demographic characteristic were obtained from the U.S. Census Bureau population and housing unit estimates for the corresponding years (*37*). A multivariable negative binomial regression model was used to assess adjusted incidence rate ratios across demographic factors (age, sex, and race) and surveillance sites (state). Chi-square tests were performed to assess the difference in proportions across two groups, and univariable negative binomial regression models were used to assess trends in the candidemia incidence rate over the 5-year surveillance period. Interaction terms between the variables in the model were examined. The tests were conducted at significance level of α = 0.05. SAS was used to perform the statistical analyses (version 9.4; SAS Institute).

### Ethical Review

CDC conducted ethical review of this surveillance activity and classified it as a nonresearch public health activity. This activity also was evaluated individually at each participating surveillance site and determined to be nonresearch in Georgia and Oregon and exempt research in Maryland. In Tennessee, the site received expedited approval from a local hospital review board, and other hospitals determined the surveillance activity to be nonresearch.

## Results

### Demographic Characteristics and Incidence

During 2012–2016, a total of 3,492 candidemia cases were identified from 3,235 patients. The median age of patients with candidemia was 59 years (interquartile range [IQR]: 45–71 years). Thirty-eight percent of patients were aged 45–64 years, and 37% were aged ≥65 years; infants aged <1 year represented 2% of cases (Table 1). Fifty-two percent were male, 45% were black, and 49% were nonblack (includes white patients, Asian patients [2%], and American Indian/Alaska Native patients [<0.05%]); race was unknown for 7%. A higher proportion of patients in Georgia and Maryland were black (56% and 59%, respectively) compared with Oregon (7%) and Tennessee (8%).

**TABLE 1:** Demographic characteristics of patients with candidemia — four sites, United States, 2012–2016

Characteristic	Georgia	Maryland	Oregon	Tennessee	Total
	(N = 1,509)	(N = 918)	(N = 334)	(N = 474)	(N = 3,235)
Median age (median, IQR)	60 (46–71)	60 (47–72)	57 (40–68)	58 (43–72)	**59 (45**–**71)**
Age group, yrs*	**No. (%)**	**No. (%)**	**No. (%)**	**No. (%)**	**No. (%)**
Infant (<1)	44 (3)	28 (3)	3 (1)	4 (1)	**79 (2)**
1–18	53 (4)	24 (3)	14 (4)	4 (1)	**95 (3)**
19–44	259 (17)	162 (18)	82 (25)	129 (27)	**632 (20)**
45–64	598 (40)	345 (38)	133 (40)	154 (32)	**1,230 (38)**
≥65	554 (37)	359 (39)	102 (31)	182 (38)	**1,197 (37)**
**Sex^†^**					
Male	786 (52)	507 (55)	162 (49)	234 (49)	**1,689 (52)**
**Race^§^**					
Black	843 (56)	539 (59)	25 (7)	36 (8)	**1,443 (45)**
White	518 (34)	337 (37)	234 (70)	421 (89)	**1,510 (47)**
American Indian/Alaska Native	2 (0)	3 (0)	6 (2)	0 (0)	**11 (0)**
Asian	34 (2)	9 (1)	10 (3)	3 (1)	**56 (2)**
Native Hawaiian/Pacific Islander	2 (0)	0 (0)	1 (0)	0 (0)	**3 (0)**

**Abbreviation:** IQR = interquartile range.

* Unknown: n = 2.

^†^ Unknown: n = 16.

^§^ Unknown: n = 212 (7%).

**FIGURE 1:**
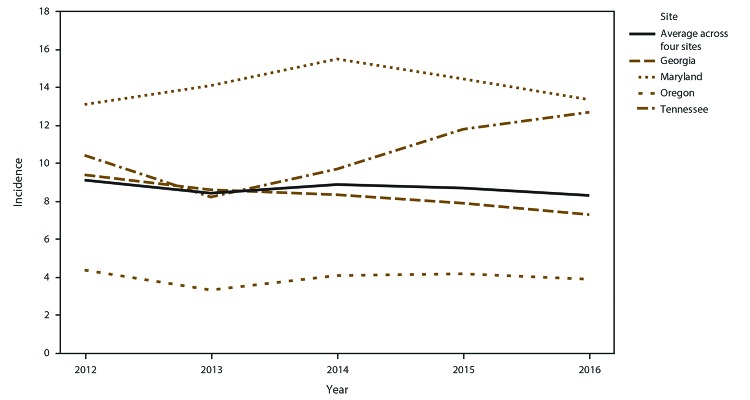
Crude annual candidemia incidence* — four sites, United States, 2012–2016 * Per 100,000 population, calculated from the U.S. Census Bureau population and housing unit estimates for the corresponding years.

The crude candidemia incidence averaged across sites and years was 8.7 per 100,000 population (range: 8.3–9.1) during 2012–2016 (Figure 1). The crude annual incidence differed by site, with the highest in Maryland (14.1 per 100,000 population) and lowest in Oregon (4.0 per 100,000 population). Adjusting for age, sex, and race, the incidence rate ratio in Maryland was 2.4 (95% confidence interval [CI]: 2.0–2.8) times the incidence in Oregon.

**FIGURE 2:**
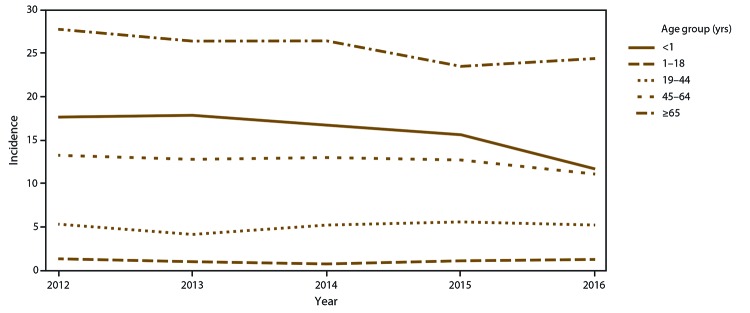
Crude annual candidemia incidence,* by age — four sites,^†^ United States, 2012–2016 * Per 100,000 population, calculated from the U.S. Census Bureau population and housing unit estimates for the corresponding years. ^†^ Georgia, Maryland, Oregon, and Tennessee.

The crude incidence of candidemia also varied by age group, with the highest crude incidence among adults aged ≥65 years (25.5 per 100,000), followed by infants aged <1 year (15.8 per 100,000). The lowest crude incidence occurred among persons aged 1–18 years (1.1 per 100,000) (Figure 2). Adjusting for sex, race, and site, the incidence rate ratio among adults aged ≥65 years was 24.2 (95% CI: 19.5–30.0) times the incidence among persons aged 1–18 years. 

**FIGURE 3:**
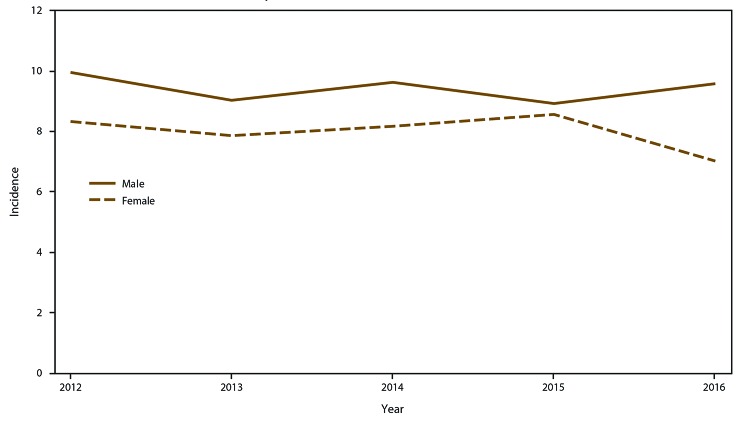
Crude annual candidemia incidence,* by sex — four sites,^†^ United States, 2012–2016 * Per 100,000 population, calculated from the U.S. Census Bureau population and housing unit estimates for the corresponding years. ^†^ Georgia, Maryland, Oregon, and Tennessee.

The crude incidence among males (9.4 per 100,000) was higher than among females (8.0 per 100,000) (Figure 3). Adjusting for age, race, and site, the candidemia incidence rate ratio among males was 1.3 (95% CI: 1.2–1.4) times the rate among females. The adjusted incidence ratio was 1.6 (95% CI: 1.2–2.3) times higher among adults aged ≥65 years.

**FIGURE 4:**
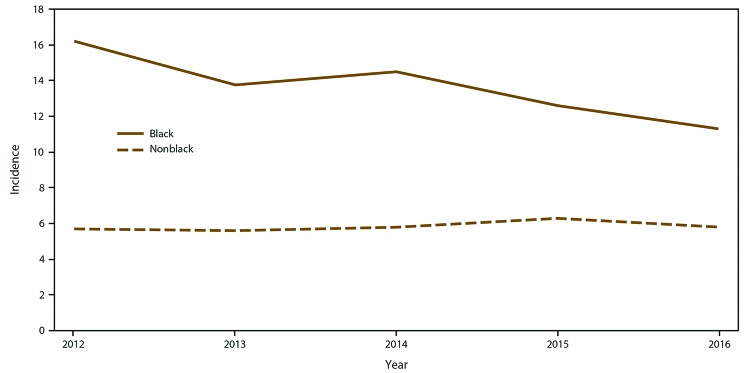
Crude annual candidemia incidence,* by race — four sites,^†^ United States, 2012–2016 * Per 100,000 population, calculated from the U.S. Census Bureau population and housing unit estimates for the corresponding years. ^†^ Georgia, Maryland, Oregon, and Tennessee.

The crude incidence among blacks was higher than among nonblacks (13.7 versus 5.8 per 100,000) (Figure 4). Adjusting for sex, age, and site, the incidence rate ratio among blacks was 2.3 (95% CI: 2.1‒2.6) times the incidence among nonblacks. The disparity in incidence by race existed across all age groups, with the adjusted incidence rate ratio ranging from 2.1 (95% CI: 1.6–2.6) times the incidence among blacks compared with nonblacks among adults aged 19–44 years to 3.1 (95% CI: 2.1–4.6) times among persons aged 1–18 years. The disparity between blacks and nonblacks persisted in all four sites, including in Georgia and Maryland, where 41%–43% of the surveillance catchment area residents were black, and in Oregon and Tennessee, where 4%–6% of catchment area residents were black. The adjusted incidence ratio comparing incidence in blacks with nonblacks ranged from 2.1 (95% CI: 1.2–3.5) in Oregon and Maryland (95% CI: 1.4–3.2) to 2.4 (95% CI: 1.6–3.7) in Georgia.

The univariable negative binomial regression estimate of the trend in incidence over the 5-year surveillance period showed no statistically significant change in incidence. No statistically significant trend in incidence over the 5-year period was found by site, age group, sex, or race (Supplementary Table, https://stacks.cdc.gov/view/cdc/80192).

### Underlying Conditions and Risk Factors for Candidemia

One third (33%) of candidemia cases were in patients with diabetes, and 17% were in patients with solid-organ malignancy. Seventeen percent were in patients with liver disease, most commonly hepatitis C virus infection (10%). Sixteen percent were in patients with chronic renal disease, and 12% were in patients who had received hemodialysis in the 90 days before the candidemia diagnosis. Three percent of cases were in patients who were infected with human immunodeficiency virus or had acquired immunodeficiency syndrome (Table 2).

**TABLE 2:** Underlying conditions and risk factors for candidemia cases — four sites, United States, 2012–2016

Underlying condition or risk factor	Georgia	Maryland	Oregon	Tennessee	Total
	(N = 1,627)	(N = 1,022)	(N = 345)	(N = 498)	(N = 3,492)
	No. (%)	No. (%)	No. (%)	No. (%)	No. (%)
Diabetes	556 (34)	372 (36)	85 (25)	139 (28)	**1,152 (33)**
Solid organ malignancy	277 (17)	194 (19)	42 (12)	83 (17)	**596 (17)**
Leukemia/lymphoma	80 (5)	55 (5)	16 (5)	19 (4)	**170 (5)**
Any liver disease	172 (11)	254 (25)	72 (21)	92 (18)	**590 (17)**
Hepatitis C	66 (4)	165 (16)	54 (16)	67 (13)	**352 (10)**
Chronic renal disease	306 (19)	199 (19)	23 (7)	30 (6)	**558 (16)**
Hemodialysis in the 90 days before candidemia diagnosis	250 (15)	142 (14)	18 (5)	24 (5)	**434 (12)**
HIV/AIDS	46 (3)	41 (4)	4 (1)	2 (0)	**93 (3)**
Injection drug use* in the last 12 months	29 (3)	68 (11)	59 (28)	45 (14)	**201 (10)**
Any surgery in the 90 days before candidemia diagnosis	557 (34)	418 (41)	73 (21)	115 (23)	**1,163 (33)**
Abdominal surgery	339 (21)	233 (23)	49 (14)	59 (12)	**680 (19)**
Solid organ or stem cell transplant	37 (2)	36 (4)	3 (1)	2 (0)	**78 (2)**
Neutropenia in the 2 days before candidemia diagnosis	59 (4)	47 (5)	21 (6)	9 (2)	**136 (4)**
Pancreatitis in the 90 days before candidemia diagnosis	43 (3)	59 (6)	6 (2)	21 (4)	**129 (4)**
Inflammatory bowel disease	20 (1)	14 (1)	3 (1)	1 (0)	**38 (1)**
Systemic antibiotics in the 14 days before candidemia diagnosis	1,268 (78)	855 (84)	235 (68)	340 (68)	**2,698 (77)**
Total parenteral nutrition in the 14 days before candidemia diagnosis	506 (31)	182 (18)	58 (17)	87 (17)	**833 (24)**
Central venous catheter in the 2 days before candidemia diagnosis	1,251 (77)	760 (74)	206 (60)	333 (67)	**2,550 (73)**
Previous hospitalization in the 90 days before candidemia diagnosis	950 (58)	591 (58)	192 (56)	275 (55)	**2,008 (58)**
Intensive care unit admission in the 14 days before or after candidemia diagnosis	953 (59)	640 (63)	139 (40)	211 (42)	**1,943 (56)**
Resident of a nursing home before hospital admission for candidemia	208 (13)	217 (21)	27 (8)	42 (8)	**494 (14)**

**Abbreviations:** AIDS = acquired immunodeficiency syndrome; HIV = human immunodeficiency virus.

* Data for this variable were not collected during 2012–2013. Numbers and percentages are for 2014–2016.

Approximately one third (33%) of cases were in patients who had a surgical procedure in the 90 days before the candidemia diagnosis; abdominal surgery (19%) was the most common type of surgery. Four percent of cases were in patients who had neutropenia in the 2 days before diagnosis. Most (77%) of cases were in patients who had received systemic antibiotics in the 14 days before diagnosis. Almost one fourth (24%) of cases were in patients who had received TPN in the 14 days before the candidemia diagnosis. Georgia had a higher proportion of cases in patients receiving TPN (31%) than other sites (17%–18%). Nearly three fourths (73%) of cases were in patients who had a CVC in place within 2 days before diagnosis. More than half (58%) of cases were in patients who had had a previous hospitalization in the 90 days before the diagnosis, and 96% were in patients who were hospitalized at the time of or in the week after the diagnosis. More than half (56%) of the cases were in patients who were in the ICU in the 14 days before or after the candidemia diagnosis (Table 2).

Ten percent of cases were in patients who had used injection drugs in the previous 12 months. The proportion of cases related to injection drug use (IDU) was higher in Oregon (28%) and Tennessee (14%) than in other sites (3% in Georgia and 11% in Maryland) (Table 2).

### Case Classification

Sixty percent of the cases were health care–onset infections, 32% were health care–associated community-onset infections, and 8% were community-onset infections (Table 3). Oregon and Tennessee had a higher proportion of community-onset cases (13%–16%) compared with Georgia and Maryland (4%–7%). The median time from admission to initial candidemia culture was 5 days (IQR: 0–16 days). The median hospital stay was 18 days (IQR: 9–35 days).

**TABLE 3:** Classification of candidemia cases, current hospitalizations, days from admission to culture, and days of hospitalization — four sites, United States, 2012–2016

Characteristics	Georgia	Maryland	Oregon	Tennessee	Total
	(N = 1,627)	(N = 1,022)	(N = 345)	(N = 498)	(N = 3,492)
**Case classification**	**No. (%)**	**No. (%)**	**No. (%)**	**No. (%)**	**No. (%)**
Health care onset*	1,064 (65)	622 (61)	170 (49)	251 (50)	**2,107 (60)**
Health care associated community onset^†^	451 (28)	358 (35)	121 (35)	183 (37)	**1,113 (32)**
Community onset^§^	112 (7)	42 (4)	54 (16)	64 (13)	**272 (8)**
**Current hospitalization for candidemia**	1,556 (96)	997 (98)	324 (94)	458 (92)	**3,335 (96)**
**Days until culture and of hospitalization**	**Median (IQR)**	**Median (IQR)**	**Median (IQR)**	**Median (IQR)**	**Median (IQR)**
Days from admission to culture	8 (1–19)	5 (0–15)	2 (0–9.5)	2 (0–11)	**5 (0–16)**
Days of hospitalization	22 (11–43)	17 (8–33)	11 (6–24.5)	12 (6–23)	**18 (9–35)**

**Abbreviation:** IQR = interquartile range.

* Initial culture positive for *Candida* was obtained ≥3 days after admission.

^†^ Culture positive for *Candida* was obtained <3 days before admission for a patient with a recent health care exposure.

^§^ Culture positive for *Candida* was obtained <3 days before admission for a patient without a recent health care exposure.

### Previous Candidemia and Previous Antifungal Treatment

Nine percent of cases occurred in patients who had a previous episode of candidemia, and the median time from previous to current candidemia episode in the same patient was 104 days (IQR: 56–253 days) (Table 4). Forty-one patients had at least three cases each of candidemia, 15 patients had at least four cases, seven patients had at least five cases, and three patients had up to six cases of candidemia. Twelve percent had received antifungal treatment in the 14 days before the candidemia diagnosis; fluconazole was the most common antifungal received before diagnosis (7%), followed by echinocandins (4%) (Table 4).

**TABLE 4:** Treatment for and outcomes of patients with candidemia cases — four sites, United States, 2012–2016

Treatment and outcome	Georgia	Maryland	Oregon	Tennessee	Total
	(N = 1,627)	(N = 1,022)	(N = 345)	(N = 498)	(N = 3,492)
**Treatment**	**No. (%)**	**No. (%)**	**No. (%)**	**No. (%)**	**No. (%)**
Previous antifungal treatment in the 14 days before candidemia	236 (15)	119 (12)	25 (7)	44 (9)	**424 (12)**
Fluconazole	160 (10)	58 (6)	5 (1)	33 (7)	**256 (7)**
Echinocandins	77 (5)	45 (4)	1 (0)	6 (1)	**129 (4)**
Amphotericin B	3 (0)	5 (0)	0 (0)	0 (0)	**8 (0)**
Other azoles*	9 (1)	9 (1)	0 (0)	0 (0)	**18 (1)**
Previous candidemia	153 (9)	123 (12)	16 (5)	27 (5)	**319 (9)**
Systemic antifungal therapy for candidemia episode^†^	1,391 (85)	834 (82)	288 (83)	363 (73)	**2,876 (82)**
Fluconazole	972 (60)	505 (49)	206 (60)	256 (51)	**1,939 (56)**
Echinocandins	888 (55)	582 (57)	137 (40)	191 (38)	**1,798 (51)**
Amphotericin B	95 (6)	54 (5)	15 (4)	9 (2)	**173 (5)**
Other azoles*	25 (2)	35 (3)	9 (3)	6 (1)	**75 (2)**
**Outcome**					
Death 48 hours after positive *Candida* culture obtained	118 (7)	87 (9)	28 (8)	56 (11)	**289 (8)**
All-cause in-hospital case-fatality ratio	394 (24)	280 (27)	69 (20)	130 (26)	**873 (25)**
Median days from positive *Candida* culture to death	**Median (IQR)**	**Median (IQR)**	**Median (IQR)**	**Median (IQR)**	**Median (IQR)**
	6 (2–14)	6 (2–14)	4 (2–13)	4 (1–11)	**6 (2**–**14)**

**Abbreviation:** IQR = interquartile range.

* Including itraconazole, posaconazole, and voriconazole.

^†^ Treatment with each class of antifungal was not mutually exclusive. Treatment might have included more than one class of antifungal.

### Antifungal Treatment

A total of 82% of 3,492 cases were treated with an antifungal for candidemia. The most common antifungal received was fluconazole (56%), followed by echinocandins (51%) (Table 4). Among cases in adults who were treated, 34% were treated with fluconazole alone and 30% with echinocandins alone; 34% received both fluconazole and echinocandins. Use of echinocandins increased over time (48% in 2012 to 55% in 2016) whereas the use of fluconazole decreased over time (57% in 2012 to 49% in 2016). Echinocandins were used more frequently in the Georgia and Maryland sites (55%–57% of cases) than in the Oregon and Tennessee sites (38%–40% of cases). Amphotericin B was primarily used to treat cases among children, with 67% of cases in infants (aged <1 year) and 31% of cases in children aged 1–18 years receiving this drug for treatment of candidemia. Of the 19% of cases in patients not receiving antifungal treatment, 33% were in patients who died within 48 hours of culture, and another 7% were in patients who were discharged to palliative care. An additional 20% were in patients who were not hospitalized or had an unknown hospitalization status, and 11% were in patients discharged before culture result was available; therefore, receipt of antifungal treatment could not be determined.

### Deaths

The all-cause in-hospital case-fatality ratio was 25% for any time after admission and 8% for <48 hours after a positive culture. The all-cause in-hospital case-fatality ratio varied by age group: 15% in infants (aged <1 year), 10% in persons aged 1–18 years, 15% in adults aged 19–44 years, 26% in adults aged 45–64 years, and 32% in adults aged ≥65 years. The median time from positive candidemia culture to death was 6 days (IQR: 2–14) (Table 4).

### Species Distribution

**FIGURE 5:**
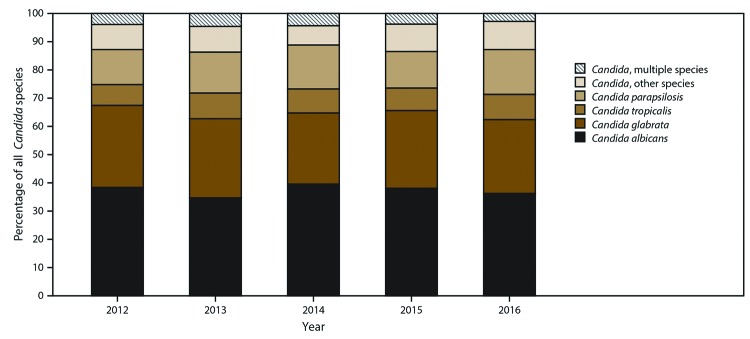
Species* distribution of *Candida* organisms, by year — four sites,^†^ United States, 2012–2016 * The category “Candida, other species” includes *C. allocifferrii, C. bracarensis, C. dubliniensis, C. fermentati, C. guilliermondii, C. kefyr, C. krusei, C. lipolytica, C. lusitaniae, C. metapsilosis, C. orthopsilosis, C. pararugosa, C. pelliculosa, C. rugosa,* and *C. sojae*. ^†^ Georgia, Maryland, Oregon, and Tennessee.

*C. albicans* accounted for 39% of cases, and other *Candida* species accounted for 61%; the most common species were *C. glabrata* (28%), *C. parapsilosis* (15%), and *Candida tropicalis* (9%). Four percent of cases involved multiple *Candida* species isolated on the date of the initial candidemia blood culture or in the 30 days after. The lowest proportion of *C. albicans* was in Maryland (35%), compared with 40%–42% in the other three sites (Figure 5; Table 5).

**TABLE 5:** Species distribution of candidemia cases — four sites, United States, 2012–2016

*Candida* species	Georgia	Maryland	Oregon	Tennessee	Total
	(N = 1,626)	(N = 1,022)	(N = 344)	(N = 498)	(N = 3,490)
	No. (%)	No. (%)	No. (%)	No. (%)	No. (%)
*C. albicans*	636 (40)	338 (35)	129 (40)	204 (42)	**1,307 (39)**
*C. glabrata*	408 (26)	309 (32)	85 (26)	147 (31)	**949 (28)**
*C. tropicalis*	120 (8)	107 (11)	17 (5)	48 (10)	**292 (9)**
*C. parapsilosis*	271 (17)	123 (13)	59 (18)	43 (9)	**496 (15)**
*C. dubliniensis*	30 (2)	44 (5)	9 (3)	18 (4)	**101 (3)**
*C. guilliermondii*	7 (0)	3 (0)	8 (3)	0 (0)	**18 (1)**
*C. lusitaniae*	41 (3)	12 (1)	2 (1)	11 (2)	**66 (2)**
*C. krusei*	36 (2)	23 (2)	9 (3)	4 (1)	**72 (2)**
*Candida*, other species*	30 (2)	10 (1)	6 (2)	7 (1)	**53 (2)**
*Candida*, multiple species	47 (3)	53 (5)	20 (5)	16 (3)	**136 (4)**

* Includes *C. allocifferrii, C. bracarensis, C. fermentati, C. kefyr, C. lipolytica, C. metapsilosis, C. orthopsilosis, C. pararugosa, C. pelliculosa, C. rugosa,* and *C. sojae.*

### Antifungal Resistance

Seven percent of the 2,997 isolates analyzed for antifungal resistance had either acquired or intrinsic resistance to fluconazole, and 1.6% were resistant to echinocandins. Fluconazole resistance was 8.6% among *C. glabrata* isolates, 7.7% among *C. parapsilosis* isolates, and 4.2% among *C. tropicalis* isolates (Table 6). Resistance to fluconazole increased from 4.4% in 2012 to 14% in 2016 among *C. parapsilosis* isolates, and no substantial increases occurred in fluconazole resistance in other species. Resistance to echinocandins varied by year for *C. glabrata* (2.1%–8.2%) and *C. albicans* (0%–0.9%). None of the *C. parapsilosis* isolates were echinocandin resistant. Multidrug resistance (i.e., resistance to two or more drug classes) was identified in 1.3% of *C. glabrata* isolates. Fluconazole resistance ranged from 5.9% to 10.3% in Georgia, 4.0% to 10.8% in Maryland, 0% to 9.6% in Oregon, and 1.6% to 8.6% in Tennessee (Table 7). Echinocandin resistance ranged from 0.4% to 4.3% in Georgia, 0.5% to 3.5% in Maryland, 0% to 1.9% in Oregon, and 0% to 2.1% in Tennessee. Multidrug resistance was only found in isolates from Georgia (0%–1.6%) and Maryland (0%–1.5%).

**TABLE 6:** Drug resistance among *Candida* isolates,* by species — four sites,^†^ United States, 2012–2016

*Candida* species and drug	2012	2013	2014	2015	2016	Total
	No. (%)	No. (%)	No. (%)	No. (%)	No. (%)	No. (%)
***C. albicans***	**N = 268**	**N = 217**	**N = 267**	**N = 246**	**N = 235**	**N = 1,233**
Amphotericin B	0	0	0	0	0	0
Fluconazole	1 (0.4)	1 (0.5)	0	2 (0.8)	0	4 (0.3)
Voriconazole	0	0	0	1 (0.4)	0	1 (0.1)
Echinocandins^§^	0	2 (0.9)	0	2 (0.8)	1 (0.4)	5 (0.4)
Multiple drugs^¶^	0	0	0	0	0	0
***C. glabrata***	**N = 205**	**N = 184**	**N = 182**	**N = 190**	**N = 168**	**N = 929**
Amphotericin B	0	0	0	0	0	0
Fluconazole	21 (10.2)	13 (7.1)	15 (8.2)	13 (6.8)	18 (10.7)	80 (8.6)
Voriconazole	—**	—	—	—	—	—
Echinocandins^§^	10 (4.9)	15 (8.2)	6 (3.3)	4 (2.1)	6 (3.6)	41 (4.4)
Multiple drugs^¶^	3 (1.5)	5 (2.7)	2 (1.1)	2 (1.1)	0	12 (1.3)
***C. krusei***	**N = 17**	**N = 12**	**N = 14**	**N = 18**	**N = 16**	**N = 77**
Amphotericin B	0	0	0	0	0	0
Fluconazole	—	—	—	—	—	—
Voriconazole	0	0	0	0	0	0
Echinocandins^§^	0	2 (16.7)	0	0	0	2 (2.6)
Multiple drugs^¶^	0	2 (16.7)	0	0	0	2 (2.6)
***C. parapsilosis***	**N = 91**	**N = 93**	**N = 108**	**N = 84**	**N = 93**	**N = 469**
Amphotericin B	0	0	0	0	0	0
Fluconazole	4 (4.4)	4 (4.3)	5 (4.6)	10 (11.9)	13 (14.0)	36 (7.7)
Voriconazole	0	2 (2.2)	1 (0.9)	3 (3.6)	4 (4.3)	10 (2.1)
Echinocandins^§^	0	0	0	0	0	0
Multiple drugs^¶^	0	0	0	0	0	0
***C. tropicalis***	**N = 52**	**N = 59**	**N = 63**	**N = 56**	**N = 59**	**N = 289**
Amphotericin B	0	0	0	0	0	0
Fluconazole	3 (5.8)	1 (1.7)	5 (7.9)	1 (1.8)	2 (3.4)	12 (4.2)
Voriconazole	1 (1.9)	1 (1.7)	2 (3.2)	0	2 (3.4)	6 (2.1)
Echinocandins^§^	0	0	0	0	0	0
Multiple drugs^¶^	0	0	0	0	0	0

* Only includes isolates sent to CDC.

^†^ Georgia, Maryland, Oregon, and Tennessee.

^§^ Defined resistant to echinocandins if an isolate was resistant to any of the echinocandins.

^¶^ Isolates were tested for resistance to any echinocandin and fluconazole.

** No breakpoints.

**TABLE 7:** Drug resistance in *Candida* isolates,* by site — four sites, United States, 2012–2016

Site and drug	2012	2013	2014	2015	2016
	No. (%)	No. (%)	No. (%)	No. (%)	No. (%)
**Georgia**	**N = 306**	**N = 253**	**N = 270**	**N = 240**	**N = 232**
Amphotericin B	0	0	0	0	0
Fluconazole	26 (8.5)	17 (6.7)	16 (5.9)	20 (8.3)	24 (10.3)
Voriconazole	1 (0.3)	1 (0.4)	2 (0.7)	0	1 (0.4)
Echinocandins^†^	6 (2.0)	11 (4.3)	5 (1.9)	1 (0.4)	3 (1.3)
Multidrug^§^	3 (1.0)	4 (1.6)	2 (0.7)	0	0
**Maryland**	**N = 186**	**N = 198**	**N = 214**	**N = 203**	**N = 177**
Amphotericin B	0	0	0	0	0
Fluconazole	10 (5.4)	8 (4.0)	16 (7.5)	22 (10.8)	19 (10.7)
Voriconazole	0	1 (0.5)	0	3 (1.5)	3 (1.7)
Echinocandins^†^	4 (2.2)	7 (3.5)	1 (0.5)	3 (1.5)	3 (1.7)
Multidrug^§^	0	3 (1.5)	0	2 (1.0)	0
**Oregon**	**N = 60**	**N = 52**	**N = 72**	**N = 54**	**N = 60**
Amphotericin B	0	0	0	0	0
Fluconazole	3 (5.0)	5 (9.6)	3 (4.2)	0	2 (3.3)
Voriconazole	0	1 (1.9)	0	0	0
Echinocandins^†^	0	1 (1.9)	0	0	0
Multidrug^§^	0	0	0	0	0
**Tennessee**	**N = 81**	**N = 62**	**N = 78**	**N = 97**	**N = 102**
Amphotericin B	0	0	0	0	0
Fluconazole	7 (8.6)	1 (1.6)	4 (5.1)	2 (2.1)	4 (3.9)
Voriconazole	0	0	1 (1.3)	1 (1.0)	2 (2.0)
Echinocandins^†^	0	0	0	2 (2.1)	1 (1.0)
Multidrug^§^	0	0	0	0	0

* Only includes isolates sent to CDC.

^†^ Defined resistant to echinocandins if an isolate was resistant to any of the echinocandins.

^§^ Isolates were tested for resistance to any echinocandin and fluconazole.

## Discussion

This report summarizes the incidence, underlying conditions, health care exposure, treatment, species distribution, antifungal resistance, and outcomes associated with approximately 3,500 candidemia cases at four CDC EIP surveillance sites during 2012–2016. The crude candidemia incidence averaged across sites and years was 8.7 per 100,000 population, and the all-cause in-hospital case-fatality ratio was 25%.

Candidemia incidence was highest in the Maryland site and lowest in the Oregon site. These rates differed significantly even after adjusting for year, race, age, and sex, suggesting that the difference cannot be fully explained by demographic characteristics over time. Unlike other pathogenic fungi, such as *Coccidioides* and *Histoplasma* species, which are more prevalent in the environment in specific geographic parts of the United States and hence result in varying incidence geographically (*38*,*39*), *Candida* is believed to be commensal in the human host. Regional differences in colonization with *Candida* in the United States have not been studied. Site-specific differences in the incidence of candidemia might be due to differences in the percentages of patients with underlying conditions such as diabetes and other immunosuppressive conditions (*40*,*41*), differences in practice patterns and use of antibiotics, and differences in use of antifungals and CVCs, all of which contribute to the risk for candidemia.

Candidemia incidence continues to be highest in adults aged ≥65 years, followed by infants aged <1 year. This contrasts with data from the early 1990s, when infants had the highest incidence (*6*), followed by substantial decreases by the late 2000s (*26*,*28*). Enhanced infection control practices, including appropriate catheter use to limit catheter-related bloodstream infections (*42*–*44*), antibiotic stewardship, and antifungal prophylaxis practices, might be responsible for some of the decreases. However, as the population of patients at risk for candidemia, such as adults aged ≥65 years or persons who are immunosuppressed, increases (*45*), other strategies to prevent candidemia in health care settings might be needed.

Candidemia incidence was higher in males than females even after adjusting for demographic factors, and this difference persisted across all adult age and race groups. Although females have more noninvasive candidiasis (primarily vaginal candidiasis) than males, invasive bloodstream infections were less common among females than among males. Although the reasons for differences in candidemia incidence by sex are unknown, these differences have been found with other fungal diseases such as paracoccidioidomycosis and coccidioidomycosis. For paracoccidioidomycosis, the differences in incidence occur in postpubertal age groups (approximately aged ≥12 years), and laboratory research has shown that estrogen levels might have a role in acquisition of fungal diseases (*46*,*47*). Whether estrogen levels or other factors play a role in differences in risk for candidemia among females and males is not well understood.

The previously reported racial disparity in candidemia persisted in this surveillance period (*6*), with a 2.3 times higher incidence among blacks than among nonblacks. This disparity was found in all surveillance sites, even though the sites had markedly different underlying population demographics (i.e., Georgia and Maryland sites, where approximately 40% of the populations in the counties under surveillance was black, compared with <10% in the Oregon and Tennessee sites [*37*]). In addition, racial disparities existed in almost all age groups. Socioeconomic factors might be a proxy for race differences and could play a role in candidemia incidence disparities (*48*,*49*). A study exploring nosocomial infections such as invasive methicillin-resistant *Staphylococcus aureus* (MRSA) infection found that racial disparity could partially be explained by socioeconomic factors such as overcrowding and limited access and availability to health care services (*50*). Differences also might exist because blacks have higher rates of diabetes, hemodialysis, and liver diseases (*51*), which are risk factors for candidemia (*52*). Additional research on the influence of race and socioeconomic factors on disparities in candidemia infections is warranted.

Known risk factors for candidemia, including diabetes, malignancies, liver and renal disease, and recent surgery, continue to be frequent among patients with candidemia. As expected, a high proportion of cases were in patients with CVCs and who received antibiotics and TPN. Although neutropenia (*53*,*54*), hematologic malignancies (*53*), and bone marrow transplants (*10*,*13*) are well-recognized risk factors for candidemia, only a small proportion (<5%) of cases were in patients with these underlying conditions. This might be due to increasing use of antifungal prophylactic regimens among patients with leukemia or lymphoma, patients who received bone marrow transplants, and chemotherapy recipients (*15*).

The finding that 10% of cases were in patients who had used injection drugs in the previous 12 months was surprising because candidemia is generally considered a health care–associated infection. Although the association between IDU and candidemia is known, IDU is not thought to be a very common contributing factor to candidemia risk. The proportion of candidemia patients with an IDU history is much higher than the estimated <1% of the entire U.S. population with a history of IDU during the previous 12 months (*55*), suggesting that those who inject drugs are at much higher risk for candidemia than the general population. Recent literature suggests IDU might be an increasingly common risk factor for candidemia (*56*). The growing opioid crisis in the United States (*57*,*58*) might be contributing to increased rates of IDU and their infectious disease sequelae (*59*). Ongoing surveillance should closely monitor trends in IDU and assess this type of drug use as an emerging risk factor for *Candida* infection and other acute infections.

Drug-resistant *Candida* species infections are a serious public health concern and were included in CDC’s 2013 Antibiotic Resistance Threat Report (*60*). *Candida* species other than *C. albicans*, which tend to be more drug resistant than *C. albicans*, accounted for 61% of isolates in the surveillance program, similar to what has been reported previously (*6*). Fluconazole resistance was fairly common in *C. glabrata* isolates; one in 10 isolates was resistant to fluconazole. Echinocandin resistance among *C. glabrata* isolates was low when taken as a whole across the surveillance program. However, as reported in a previous publication using EIP surveillance data, resistance tends to be concentrated in a few tertiary care hospitals that care for high-acuity patients with malignancies and transplants; three hospitals out of 80 included in the candidemia EIP surveillance accounted for more than half of all echinocandin-resistant isolates (*19*). Although a concern for echinocandin resistance in *C. parapsilosis* exists because of a naturally occurring variation in the protein target for echinocandins (*61*), no echinocandin resistance was identified among *C. parapsilosis* isolates in this surveillance program. Nevertheless, increasing fluconazole resistance was noted among *C. parapsilosis* isolates. Clinicians who treat patients with candidemia should strongly consider obtaining antifungal susceptibility testing (AFST) and be aware of local antifungal resistance patterns when making treatment decisions.

Species-level identification and AFST are important aspects of candidemia management. However, availability of both types of testing, especially AFST, is limited in clinical laboratories (*62*). Availability is improving through expansion of new types of species identification methods such as MALDI-TOF and the establishment of CDC’s Antibiotic Resistance Laboratory Network (*63*), which conducts fungal species identification and tests for antifungal susceptibility.

In contrast with the 2009 Infectious Disease Society of America guidelines for the treatment of invasive candidiasis, in which echinocandins were recommended only for neutropenic patients and patients with previous exposure to antifungals (*64*), the 2016 guidelines recommend echinocandins as the initial therapy for treatment of most types of invasive candidiasis among adults (*43*). The change in recommendations was based on the increasing frequency of infections caused by species other than *C. albicans*, increasing levels of fluconazole resistance, and evidence that echinocandins are more effective. Echinocandin use before 2016 increased, and changes in practice can sometimes precede updates in guidelines. As echinocandins are used with greater frequency, continuing to monitor both trends in treatment patterns as well as resistance to echinocandins is important. Resistance to echinocandins will be problematic because of the limited antifungal armamentarium. Limited alternatives that do exist (such as amphotericin B) have substantial toxicity (*65*). Health care facilities should consider assessing antifungal use as part of antimicrobial stewardship programs to help preserve treatment options for the future.

Although cases of *C. auris* were not detected in the surveillance sites during 2012–2016, ongoing transmission of *C. auris* has been detected in several areas in the United States, primarily in Illinois, New Jersey, and New York (*66*), posing an emerging threat in the United States and worldwide because of high-level antifungal resistance and spread in health care facilities (*67*–*69*). As of July 2019, approximately 700 clinical cases of *C. auris* had been documented in the United States (*22*). Infections with other rare and drug-resistant *Candida* species, including *Candida haemulonii*, *Candida duobushaemulonii*, and *Candida rugosa*, have been reported from surveillance in other countries (*70*,*71*). Ongoing surveillance for infections caused by *Candida* species will be critical in detecting rare and emerging drug-resistant species in the United States before they become widespread.

## Limitations

The findings in this report are subject to at least four limitations. First, underlying conditions and predisposing factors described in this report were extracted from medical charts, which might have resulted in underestimates of certain conditions, such as IDU, which might not be systematically recorded on medical charts. Second, although the surveillance was active, population based, and frequently audited, certain culture-proven cases might have been missed, likely underestimating the number of infections. In addition, this surveillance underestimates the true proportion of invasive candidiasis because it only includes cases positive by blood culture, which has suboptimal sensitivity, particularly for intraabdominal candidiasis, or infections in which blood cultures were not obtained. Third, surveillance data were available from 22 counties in four states representing 2.5% of the U.S. population and therefore are not nationally representative. Finally, only five time points were assessed, which limits the ability to understand long-term trends. Nevertheless, data presented in this report describe surveillance information on geographically and demographically diverse populations and are the largest data source of population-based candidemia incidence data in the United States.

## Conclusion

Candidemia remains a serious cause of illness and death in the United States, and surveillance data are necessary to focus prevention efforts. Active surveillance for candidemia should continue to monitor incidence trends by age and race, track emergence of resistance and species distribution, monitor changes in underlying conditions and predisposing factors, and assess trends in antifungal treatment and outcomes. Surveillance was expanded to nine sites in 2017, and ongoing surveillance efforts are expected to improve the development of treatment and prevention efforts.

## References

[1] Pfaller MA, Diekema DJ. Epidemiology of invasive candidiasis: a persistent public health problem. Clin Microbiol Rev 2007;20:133–63. 10.1128/CMR.00029-06 17223626 PMC1797637

[2] Vallabhaneni S, Mody RK, Walker T, Chiller T. The global burden of fungal diseases. Infect Dis Clin North Am 2016;30:1–11. 10.1016/j.idc.2015.10.004 26739604

[3] Magill SS, O’Leary E, Janelle SJ, ; Emerging Infections Program Hospital Prevalence Survey Team. Changes in prevalence of health care-associated infections in U.S. hospitals. N Engl J Med 2018;379:1732–44. 10.1056/NEJMoa1801550 30380384 PMC7978499

[4] Wisplinghoff H, Bischoff T, Tallent SM, Seifert H, Wenzel RP, Edmond MB. Nosocomial bloodstream infections in U.S. hospitals: analysis of 24,179 cases from a prospective nationwide surveillance study. Clin Infect Dis 2004;39:309–17. 10.1086/421946 15306996

[5] Weiner LM, Webb AK, Limbago B, Antimicrobial-resistant pathogens associated with healthcare-associated infections: summary of data reported to the National Healthcare Safety Network at the Centers for Disease Control and Prevention, 2011–2014. Infect Control Hosp Epidemiol 2016;37:1288–301. 10.1017/ice.2016.174 27573805 PMC6857725

[6] Cleveland AA, Farley MM, Harrison LH, Changes in incidence and antifungal drug resistance in candidemia: results from population-based laboratory surveillance in Atlanta and Baltimore, 2008–2011. Clin Infect Dis 2012;55:1352–61. 10.1093/cid/cis697 22893576 PMC4698872

[7] Nucci M, Anaissie E. Revisiting the source of candidemia: skin or gut? Clin Infect Dis 2001;33:1959–67. 10.1086/323759 11702290

[8] Sims CR, Ostrosky-Zeichner L, Rex JH. Invasive candidiasis in immunocompromised hospitalized patients. Arch Med Res 2005;36:660–71. 10.1016/j.arcmed.2005.05.015 16216647

[9] Wey SB, Mori M, Pfaller MA, Woolson RF, Wenzel RP. Risk factors for hospital-acquired candidemia. A matched case-control study. Arch Intern Med 1989;149:2349–53. 10.1001/archinte.1989.00390100145030 2802900

[10] Kullberg BJ, Arendrup MC. Invasive candidiasis. N Engl J Med 2015;373:1445–56. 10.1056/NEJMra1315399 26444731

[11] Pammi M, Holland L, Butler G, Gacser A, Bliss JM. *Candida parapsilosis* is a significant neonatal pathogen: a systematic review and meta-analysis. Pediatr Infect Dis J 2013;32:e206–16. 10.1097/INF.0b013e3182863a1c 23340551 PMC3681839

[12] Feja KN, Wu F, Roberts K, Risk factors for candidemia in critically ill infants: a matched case-control study. J Pediatr 2005;147:156–61. 10.1016/j.jpeds.2005.02.021 16126040 PMC2031014

[13] Pappas PG, Lionakis MS, Arendrup MC, Ostrosky-Zeichner L, Kullberg BJ. Invasive candidiasis. Nat Rev Dis Primers 2018;4:18026. 10.1038/nrdp.2018.26 29749387

[14] Schelenz S, Hagen F, Rhodes JL, First hospital outbreak of the globally emerging *Candida auris* in a European hospital. Antimicrob Resist Infect Control 2016;5:35. 10.1186/s13756-016-0132-5 27777756 PMC5069812

[15] Segal BH, Almyroudis NG, Battiwalla M, Prevention and early treatment of invasive fungal infection in patients with cancer and neutropenia and in stem cell transplant recipients in the era of newer broad-spectrum antifungal agents and diagnostic adjuncts. Clin Infect Dis 2007;44:402–9. 10.1086/510677 17205448

[16] Kaufman D, Boyle R, Hazen KC, Patrie JT, Robinson M, Donowitz LG. Fluconazole prophylaxis against fungal colonization and infection in preterm infants. N Engl J Med 2001;345:1660–6. 10.1056/NEJMoa010494 11759644

[17] Lockhart SR, Iqbal N, Cleveland AA, Species identification and antifungal susceptibility testing of *Candida* bloodstream isolates from population-based surveillance studies in two U.S. cities from 2008 to 2011. J Clin Microbiol 2012;50:3435–42. 10.1128/JCM.01283-12 22875889 PMC3486211

[18] Dimopoulos G, Ntziora F, Rachiotis G, Armaganidis A, Falagas ME. *Candida albicans* versus non-*albicans* intensive care unit-acquired bloodstream infections: differences in risk factors and outcome. Anesth Analg 2008;106:523–9. 10.1213/ane.0b013e3181607262 18227310

[19] Vallabhaneni S, Cleveland AA, Farley MM, Epidemiology and risk factors for echinocandin nonsusceptible *Candida glabrata* bloodstream infections: data from a large multisite population-based candidemia surveillance program, 2008–2014. Open Forum Infect Dis 2015;2:ofv163. 26677456 10.1093/ofid/ofv163 PMC4677623

[20] Alexander BD, Johnson MD, Pfeiffer CD, Increasing echinocandin resistance in *Candida glabrata*: clinical failure correlates with presence of FKS mutations and elevated minimum inhibitory concentrations. Clin Infect Dis 2013;56:1724–32. 10.1093/cid/cit136 23487382 PMC3658363

[21] Satoh K, Makimura K, Hasumi Y, Nishiyama Y, Uchida K, Yamaguchi H. *Candida auris* sp. nov., a novel Ascomycetous yeast isolated from the external ear canal of an inpatient in a Japanese hospital. Microbiol Immunol 2009;53:41–4. 10.1111/j.1348-0421.2008.00083.x 19161556

[22] CDC. Tracking *Candida auris* [Internet]. Atlanta, GA: US Department of Health and Human Services, CDC; 2018. https://www.cdc.gov/fungal/diseases/candidiasis/tracking-c-auris.html

[23] Banerjee SN, Emori TG, Culver DH, National Nosocomial Infections Surveillance System. Secular trends in nosocomial primary bloodstream infections in the United States, 1980–1989. Am J Med 1991;91(3B):86S–9S. 10.1016/0002-9343(91)90349-3 1928197

[24] Gaynes RP, Culver DH, Emori TG, The national nosocomial infections surveillance system: plans for the 1990s and beyond. Am J Med 1991;91(3B):116S–20S. 10.1016/0002-9343(91)90355-2 1656746

[25] Fridkin SK, Kaufman D, Edwards JR, Shetty S, Horan T. Changing incidence of *Candida* bloodstream infections among NICU patients in the United States: 1995–2004. Pediatrics 2006;117:1680–7. 10.1542/peds.2005-1996 16651324

[26] Benedict K, Roy M, Kabbani S, Neonatal and pediatric candidemia: results from population-based active laboratory surveillance in four U.S. locations, 2009–2015. J Pediatric Infect Dis Soc 2018;7:e78–85 . 10.1093/jpids/piy009 29522195

[27] Fisher BT, Ross RK, Localio AR, Prasad PA, Zaoutis TE. Decreasing rates of invasive candidiasis in pediatric hospitals across the United States. Clin Infect Dis 2014;58:74–7. 10.1093/cid/cit679 24114736

[28] Cleveland AA, Harrison LH, Farley MM, Declining incidence of candidemia and the shifting epidemiology of *Candida* resistance in two U.S. metropolitan areas, 2008–2013: results from population-based surveillance. PLoS One 2015;10:e0120452. 10.1371/journal.pone.0120452 25822249 PMC4378850

[29] Kao AS, Brandt ME, Pruitt WR, The epidemiology of candidemia in two United States cities: results of a population-based active surveillance. Clin Infect Dis 1999;29:1164–70. 10.1086/313450 10524958

[30] Hajjeh RA, Sofair AN, Harrison LH, Incidence of bloodstream infections due to *Candida* species and in vitro susceptibilities of isolates collected from 1998 to 2000 in a population-based active surveillance program. J Clin Microbiol 2004;42:1519–27. 10.1128/JCM.42.4.1519-1527.2004 15070998 PMC387610

[31] CDC. *Candida auris.* 2019 case definition [Internet]. Atlanta, GA: US Department of Health and Human Services, CDC; 2019. https://wwwn.cdc.gov/nndss/conditions/candida-auris/case-definition/2019

[32] CDC. Emerging Infections Program [Internet]. Atlanta, GA: US Department of Health and Human Services, CDC; 2018. https://www.cdc.gov/ncezid/dpei/eip/index.html

[33] Deak E, Etienne KA, Lockhart SR, Gade L, Chiller T, Balajee SA. Utility of a Luminex-based assay for multiplexed, rapid species identification of *Candida* isolates from an ongoing candidemia surveillance. Can J Microbiol 2010;56:348–51. 10.1139/W10-003 20453902

[34] Hillenkamp F, Karas M, Beavis RC, Chait BT. Matrix-assisted laser desorption/ionization mass spectrometry of biopolymers. Anal Chem 1991;63:1193A–203A. 10.1021/ac00024a716 1789447

[35] Clinical and Laboratory Standards Institute (CLSI). Reference method for broth dilution antifungal susceptibility testing of yeasts; approved standard—third edition [Internet]. CLSI document no. M27–A3. Vol. 27, No. 9. Wayne, PA: Clinical and Laboratory Standards Institute; 2008. https://clsi.org/media/1461/m27a3_sample.pdf

[36] Pfaller MA, Espinel-Ingroff A, Canton E, Wild-type MIC distributions and epidemiological cutoff values for amphotericin B, flucytosine, and itraconazole and *Candida* spp. as determined by CLSI broth microdilution. J Clin Microbiol 2012;50:2040–6. 10.1128/JCM.00248-12 22461672 PMC3372147

[37] US Census Bureau. Population and housing unit estimates data. Washington, DC: US Census Bureau; 2018. https://www.census.gov/programs-surveys/popest/data.html

[38] Freedman M, Jackson BR, McCotter O, Benedict K. Coccidioidomycosis outbreaks, United States and worldwide, 1940–2015. Emerg Infect Dis 2018;24:417–23. 10.3201/eid2403.170623 29460741 PMC5823332

[39] Armstrong PA, Jackson BR, Haselow D, Multistate epidemiology of histoplasmosis, United States, 2011–2014. Emerg Infect Dis 2018;24:425–31. 10.3201/eid2403.171258 29460731 PMC5823339

[40] Mokdad AH, Ballestros K, Echko M, ; US Burden of Disease Collaborators. The state of U.S. health, 1990–2016: burden of diseases, injuries, and risk factors among U.S. states. JAMA 2018;319:1444–72. 10.1001/jama.2018.0158 29634829 PMC5933332

[41] Murray CJL, Kulkarni SC, Michaud C, Eight Americas: investigating mortality disparities across races, counties, and race-counties in the United States. PLoS Med 2006;3:e260. 10.1371/journal.pmed.0030260 16968116 PMC1564165

[42] Sanchez GV, Fleming-Dutra KE, Roberts RM, Hicks LA. Core elements of outpatient antibiotic stewardship. MMWR Recomm Rep 2016;65(No. RR-6). 10.15585/mmwr.rr6506a1 27832047

[43] Pappas PG, Kauffman CA, Andes DR, Clinical practice guideline for the management of candidiasis: 2016 update by the Infectious Diseases Society of America. Clin Infect Dis 2016;62:e1–e50. 10.1093/cid/civ1194 26679628 PMC4725385

[44] Waters TM, Daniels MJ, Bazzoli GJ, Effect of Medicare’s nonpayment for hospital-acquired conditions: lessons for future policy. JAMA Intern Med 2015;175:347–54. 10.1001/jamainternmed.2014.5486 25559166 PMC5508870

[45] Lamoth F, Lockhart SR, Berkow EL, Calandra T. Changes in the epidemiological landscape of invasive candidiasis. J Antimicrob Chemother 2018;73(suppl_1):i4–13. 10.1093/jac/dkx444 29304207 PMC11931512

[46] Restrepo A, Salazar ME, Cano LE, Stover EP, Feldman D, Stevens DA. Estrogens inhibit mycelium-to-yeast transformation in the fungus *Paracoccidioides brasiliensis*: implications for resistance of females to paracoccidioidomycosis. Infect Immun 1984;46:346–53. 6500694 10.1128/iai.46.2.346-353.1984 PMC261537

[47] Salazar ME, Restrepo A, Stevens DA. Inhibition by estrogens of conidium-to-yeast conversion in the fungus *Paracoccidioides brasiliensis*. Infect Immun 1988;56:711–3. 3343055 10.1128/iai.56.3.711-713.1988 PMC259352

[48] Rees JR, Pinner RW, Hajjeh RA, Brandt ME, Reingold AL. The epidemiological features of invasive mycotic infections in the San Francisco Bay area, 1992–1993: results of population-based laboratory active surveillance. Clin Infect Dis 1998;27:1138–47. 10.1093/clinids/27.5.1138 9827260

[49] Strollo S, Lionakis MS, Adjemian J, Steiner CA, Prevots DR. Epidemiology of hospitalizations associated with invasive candidiasis, United States, 2002–2012. Emerg Infect Dis 2016;23:7–13. 10.3201/eid2301.161198 27983497 PMC5176241

[50] See I, Wesson P, Gualandi N, Socioeconomic factors explain racial disparities in invasive community-associated methicillin-resistant *Staphylococcus aureus* disease rates. Clin Infect Dis 2017;64:597–604. 10.1093/cid/ciw808 28362911 PMC5656382

[51] Wong MD, Shapiro MF, Boscardin WJ, Ettner SL. Contribution of major diseases to disparities in mortality. N Engl J Med 2002;347:1585–92. 10.1056/NEJMsa012979 12432046

[52] Fanfair RN. Blacks at twofold increased risk for candidemia infections. Infectious Disease News, Science Daily. Rockville, MD; 2011. https://www.healio.com/infectious-disease/vaccine-preventable-diseases/news/print/infectious-disease-news/%7Bf5afa7f7-85d7-4be9-9700-d02625b9c821%7D/blacks-at-twofold-increased-risk-for-candidemia-infections

[53] Alp S, Arikan-Akdagli S, Gulmez D, Ascioglu S, Uzun O, Akova M. Epidemiology of candidaemia in a tertiary care university hospital: 10-year experience with 381 candidaemia episodes between 2001 and 2010. Mycoses 2015;58:498–505. 10.1111/myc.12349 26155849

[54] Fraser VJ, Jones M, Dunkel J, Storfer S, Medoff G, Dunagan WC. Candidemia in a tertiary care hospital: epidemiology, risk factors, and predictors of mortality. Clin Infect Dis 1992;15:414–21. 10.1093/clind/15.3.414 1520786

[55] Lansky A, Finlayson T, Johnson C, Estimating the number of persons who inject drugs in the United States by meta-analysis to calculate national rates of HIV and hepatitis C virus infections. PLoS One 2014;9:e97596. 10.1371/journal.pone.0097596 24840662 PMC4026524

[56] Poowanawittayakom N, Dutta A, Stock S, Touray S, Ellison RT 3rd, Levitz SM. Reemergence of intravenous drug use as risk factor for candidemia, Massachusetts, USA. Emerg Infect Dis 2018;24:631–7. 10.3201/eid2404.171807 29553923 PMC5875264

[57] Vivolo-Kantor AM, Seth P, Gladden RM, Trends in emergency department visits for suspected opioid overdoses—United States, July 2016–September 2017. MMWR Morb Mortal Wkly Rep 2018;67:279–85. 10.15585/mmwr.mm6709e1 29518069 PMC5844282

[58] Seth P, Scholl L, Rudd RA, Bacon S. Overdose deaths involving opioids, cocaine, and psychostimulants—United States, 2015–2016. MMWR Morb Mortal Wkly Rep 2018;67:349–58. 10.15585/mmwr.mm6712a1 29596405 PMC5877356

[59] Jackson KA, Bohm MK, Brooks JT, Invasive methicillin-resistant *Staphylococcus aureus* infections among persons who inject drugs—six sites, 2005–2016. MMWR Morb Mortal Wkly Rep 2018;67:625–8. 10.15585/mmwr.mm6722a2 29879096 PMC5991809

[60] CDC. Antibiotic resistance threats in the United States. Washington, DC: US Department of Health and Human Services; 2013. https://www.cdc.gov/drugresistance/threat-report-2013/index.html

[61] Perlin DS. Echinocandin resistance in *Candida*. Clin Infect Dis 2015;61(Suppl 6):S612–7. 10.1093/cid/civ791 26567278 PMC4643482

[62] Vallabhaneni S, Sapiano M, Weiner LM, Lockhart SR, Magill S. Antifungal susceptibility testing practices at acute care hospitals enrolled in the National Healthcare Safety Network, United States, 2011–2015. Open Forum Infect Dis 2017;4:ofx175. 10.1093/ofid/ofx175 29026868 PMC5632525

[63] CDC. Lab capacity: Antibiotic Resistance Laboratory Network (AR Lab Network) [Internet]. Atlanta, GA: US Department of Health and Human Services, CDC; 2018. https://www.cdc.gov/drugresistance/solutions-initiative/ar-lab-networks.html

[64] Pappas PG, Kauffman CA, Andes D, Infectious Diseases Society of America. Clinical practice guidelines for the management of candidiasis: 2009 update by the Infectious Diseases Society of America. Clin Infect Dis 2009;48:503–35. 10.1086/596757 19191635 PMC7294538

[65] Fanos V, Cataldi L. Amphotericin B-induced nephrotoxicity: a review. J Chemother 2000;12:463–70. 10.1179/joc.2000.12.6.463 11154026

[66] Chow NA, Gade L, Tsay SV, US *Candida auris* Investigation Team. Multiple introductions and subsequent transmission of multidrug-resistant *Candida auris* in the USA: a molecular epidemiological survey. Lancet Infect Dis 2018;18:1377–84. 10.1016/S1473-3099(18)30597-8 30293877 PMC6556114

[67] Lockhart SR, Etienne KA, Vallabhaneni S, Simultaneous emergence of multidrug-resistant *Candida auris* on 3 continents confirmed by whole-genome sequencing and epidemiological analyses. Clin Infect Dis 2017;64:134–40. 10.1093/cid/ciw691 27988485 PMC5215215

[68] Welsh RM, Bentz ML, Shams A, Survival, persistence, and isolation of the emerging multidrug-resistant pathogenic yeast *Candida auris* on a plastic health care surface. J Clin Microbiol 2017;55:2996–3005. 10.1128/JCM.00921-17 28747370 PMC5625385

[69] Vallabhaneni S, Kallen A, Tsay S, . Investigation of the first seven reported cases of *Candida auris*, a globally emerging invasive, multidrug-resistant fungus—United States, May 2013–August 2016. MMWR Morb Mortal Wkly Rep 2016;65:1234–7. 27832049 10.15585/mmwr.mm6544e1

[70] Ramos R, Caceres DH, Perez M, Red Nacional de Vigilancia Epidemiologica en Microbiologia Clinica. Emerging multidrug-resistant *Candida duobushaemulonii* infections in Panama hospitals: importance of laboratory surveillance and accurate identification. J Clin Microbiol 2018;56:e00371–18. 29695521 10.1128/JCM.00371-18 PMC6018349

[71] Escandón P, Cáceres DH, Espinosa-Bode A, Notes from the field: surveillance for *Candida auris*—Colombia, September 2016–May 2017. MMWR Morb Mortal Wkly Rep 2018;67:459–60. 29672473 10.15585/mmwr.mm6715a6 PMC6191104

