# *Erratum:* Vol. 68, No. SS-8

**DOI:** 10.15585/mmwr.mm6841a2

**Published:** 2019-10-18

In the *MMWR* Surveillance Summary “Population-Based Active Surveillance for Culture-Confirmed Candidemia — Four Sites, United States, 2012–2016,” on page 9, the second and third footnotes (^†^ and ^§^) for Table 3 should have read

^†^ Culture positive for *Candida *was obtained <3 days **after** admission for a patient with a recent health care exposure.

^§^ Culture positive for *Candida *was obtained <3 days **after** admission for a patient without a recent health care exposure.

